# Role of Crosstalk between Glial Cells and Immune Cells in Blood-Brain Barrier Damage and Protection after Acute Ischemic Stroke

**DOI:** 10.14336/AD.2023.1010

**Published:** 2023-10-13

**Authors:** Yihui Wang, Wencao Liu, Panpan Geng, Weihong Du, Chun Guo, Qian Wang, Guo-qing Zheng, Xinchun Jin

**Affiliations:** ^1^Beijing Key Laboratory of Cancer Invasion and Metastasis Research, Department of Histology and Embryology, School of Basic Medical Sciences, Advanced Innovation Center for Human Brain Protection, Capital Medical University, Beijing, 100069, China.; ^2^Shanxi Provincial People's Hospital, Taiyuan 030001, China.; ^3^School of Biosciences, University of Sheffield, Firth Court, Western Bank, Sheffield, UK.; ^4^Department of Neurology, The First Affiliated Hospital of Zhejiang Chinese Medical University (Zhejiang Provincial Hospital of Chinese Medicine), Hangzhou, China

**Keywords:** acute ischemic stroke, blood-brain barrier, glial cells, immune cells, inflammation

## Abstract

Blood-brain barrier (BBB) damage is the main pathological basis for acute ischemic stroke (AIS)-induced cerebral vasogenic edema and hemorrhagic transformation (HT). Glial cells, including microglia, astrocytes, and oligodendrocyte precursor cells (OPCs)/oligodendrocytes (OLs) play critical roles in BBB damage and protection. Recent evidence indicates that immune cells also have an important role in BBB damage, vasogenic edema and HT. Therefore, regulating the crosstalk between glial cells and immune cells would hold the promise to alleviate AIS-induced BBB damage. In this review, we first introduce the roles of glia cells, pericytes, and crosstalk between glial cells in the damage and protection of BBB after AIS, emphasizing the polarization, inflammatory response and crosstalk between microglia, astrocytes, and other glia cells. We then describe the role of glial cell-derived exosomes in the damage and protection of BBB after AIS. Next, we specifically discuss the crosstalk between glial cells and immune cells after AIS. Finally, we propose that glial cells could be a potential target for alleviating BBB damage after AIS and we discuss some molecular targets and potential strategies to alleviate BBB damage by regulating glial cells after AIS.

## 1. Introduction

Impairment of the BBB is the main pathological basis of HT and vasogenic edema following acute ischemic stroke (AIS) and treatment with tissue plasminogen activator (tPA), the only FDA-approved thrombolytic drug after AIS[1]. Multiple cascade changes are implicated in BBB damage, such as inflammation, immune responses, tight junction proteins (TJPs) degradation, and trans-endothelial hyperpermeability [2, 3]. Of note, the endothelial cells (ECs), glial cells, inflammatory factors, and immune cells are all involved in immune responses after AIS, where immune cells are quickly activated and migrate to the ischemic hemisphere, where they continue to influence the progress and prognosis [4].

Glial cells, including microglia, astrocytes, oligodendrocytes (OLs), and oligodendrocyte precursor cells (OPCs), are a major component of the peri-infarct environment in the central nervous system (CNS). They are involved in immune regulation after AIS and have emerged as promising therapeutic targets for AIS [5]. Glial cells play both beneficial and detrimental roles after AIS [6]. For example, microglia, which are rapidly activated after AIS[7], monitor CNS homeostasis, regulate innate immune responses, and actively communicate with ECs to regulate BBB integrity [8]. Microglia can express both pro-inflammatory and anti-inflammatory phenotypic markers after AIS [9]. Activated microglia can secrete anti-inflammatory cytokines and neurotrophin, which are beneficial to the recovery of BBB [10]. In contrast, pro-inflammatory microglia secreted pro-inflammatory cytokines, such as interleukins and chemokines, can increase BBB permeability and TNF-α can induce ECs necroptosis after AIS [11]. In addition, reactive astrocytes secrete pro-inflammatory cytokines leading to secondary brain damage and neurotrophin to protect the brain and promote brain remodeling [12, 13]. Furthermore, previous studies have been shown that OPCs transplantation can significantly reduce infarct volume and brain edema, protect the integrity of BBB, and promote neurological functional recovery after AIS in mice [14], and OPCs treatment can upregulate TJPs after AIS via activating Wnt/β-catenin pathway [15].

There is growing evidence that glial cell activation after AIS is important for maintaining the integrity of BBB. In the current review, we first introduce the role of glial cells, pericytes and crosstalk between glial cells in the damage and protection of BBB after AIS. We then describe the role of glial cell-derived exosomes in the damage and protection of BBB after AIS. Next, we further discuss the crosstalk between glial cells and peripheral immune cells after AIS. Finally, we propose that glial cells could be a therapeutic target for alleviating BBB damage after AIS.

**Figure 1.**
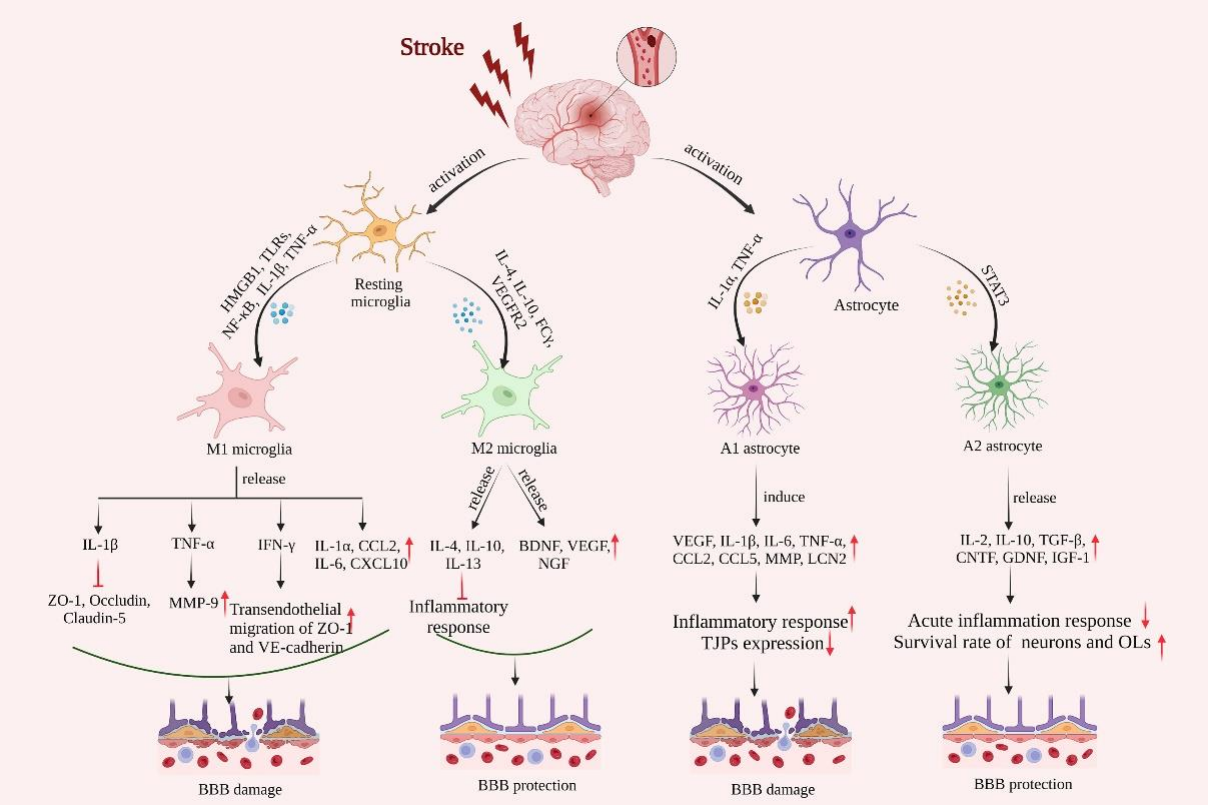
**Schematic representation of activation of microglia and astrocytes and their effects on the integrity of the BBB after AIS**. Resting microglia and astrocytes are activated and polarized into M1/M2 microglia and A1/A2 astrocytes. M1 microglia promote the production of pro-inflammatory cytokines such as interleukin-1β (IL-1β), tumor necrosis factor-α (TNF-α), interferon-γ (IFN-γ), interleukin-1α (IL-1α), interleukin-6 (IL-6), CCL2, and C-X-C motif chemokine ligand 10 (CXCL10). IL-1β inhibits the expression of tight junction proteins (TJPs), TNF-α increases the expression of matrix metalloproteinase-9 (MMP9), and IFN-γ enhances the transendothelial migration of ZO-1 and VE-cadherin. M2 microglia release growth and trophic factors such as BDNF, vascular endothelial growth factor (VEGF), nerve growth factor (NGF), and anti-inflammatory cytokines such as interleukin-4 (IL-4), interleukin-10 (IL-10) and interleukin-13 (IL-13) that inhibit inflammatory responses and enhance BBB protection. A1-type astrocytes induce pro-inflammatory production of cytokines such as IL-1β, IL-6, TNF-α, CCL2, C-C chemokine ligand 5 (CCL5), and MMP, Lipocalin-2 (LCN2), enhancing the inflammatory response and expression of TJPs leading to BBB damage. A2-type astrocytes release growth and trophic factors such as ciliary neurotrophic factor, glia-derived neurotrophic factor (GDNF), insulin-like growth factor 1 (IGF-1) and transforming growth factor-β (TGF-β) and anti-inflammatory cytokines such as interleukin-2 (IL-2) and IL-10, protect the BBB by reducing acute inflammation and increasing neuronal and oligodendrocyte (OL) survival.

## 2. Physiological structure and function of BBB

BBB is mainly composed of ECs, pericytes, astrocytes, extracellular matrix (ECM), and basement membrane (BM)[16]. ECs have continuous intercellular TJPs, the front line of the BBB [17]. The impaired function of TJPs in ECs leads to deficient BBB integrity, which can cause swelling or neurotoxicity[18]. Astrocytes, ECs, and pericytes contribute to the formation of BM by secreting ECM. The BM mainly consists of collagen, laminin, and other glycoproteins [19]. BM regulates intercellular communication and BBB function by interacting with ECM [20].

Pericytes can not only control BBB permeability and cerebral blood flow [21, 22], but also have a high phagocytic activity [23]. Astrocytes specifically express intermediate filaments vimentin and glial fibrillary acidic protein (GFAP) [24]. Proper regulation of astrocyte function is considered to be the key to enhanced BBB function and diminished BBB damage after AIS because astrocytes have endfeet that tightly wrap around the vessel wall, cover almost the entire surface of the brain capillaries, and are essential for the induction and maintenance of the TJPs barrier [25]. Microglia are resident immune cells in the CNS that screen the brain parenchyma for blood-borne substances and potentially inflammatory stimuli, providing a front line of defense. Notably, there is evidence that activated microglia can regulate the expression of TJPs, thereby improving the integrity of the BBB [26].

## 3. Role of crosstalk between glial cells in the damage and protection of BBB after AIS (Fig. 1)

### 3.1 Polarization of microglia and crosstalk between microglia and other glial cells

Microglia typically exhibit a ramified morphology with multiple radial projections extending from the cell body, characterized by smaller soma. The cells exhibit endocytosis, phagocytosis, and limited cell motility and are often referred to as "resting state" [27]. After AIS, inflammation occurs following the production of reactive oxygen species (ROS), cell necrosis, and tissue damage, which in turn activates resident immune cells [28]. Activated microglia exhibit morphological changes such as hypertrophic cell body [29] and have been classified into different phenotypes: M1-type microglia that produce pro-inflammatory cytokines, and M2-type microglia that generate proangiogenic and anti-inflammatory cytokines [30]. The response of microglia to AIS is dynamic due to the different states of acute, subacute, and chronic recovery periods after AIS [31, 32]. Shortly after AIS, the microglia that initially infiltrated the infarcted area were the M2 phenotype [30]. However, the response of M2-type microglia was extremely transient, with a gradual increase reaching a peak by 1 to 3 days after middle cerebral artery occlusion (MCAO) and a downregulation at 7 days. At 3 days post-MCAO, the number of M1-type microglia increased in the injured area and accompanied by an increase in the secretion of pro-inflammatory cytokines, such as TNF-α and NO, and the expression of the M2 phenotype protein was suppressed [33].

More importantly, a variety of factors affect microglia activation after AIS, including toll-like receptors (TLRs), especially TLR4, high-mobility group box 1 (HMGB1), chemokine and cytokine receptors, purinergic receptors, and glutamate receptors. These factors are involved in a variety of signaling pathways, such as mitogen-activated protein kinase cascades, NF-κb, and peroxisome proliferator-activated receptors (PPARs) [34, 35]. TLR4, expressed on the surface of microglia, repeatedly recognizes different pathogen-associated molecular patterns via extracellular leucine, ultimately leading to NF-κB production and increased release of pro-inflammatory factors [36]. After AIS, NF-κB p65 and p50 form heterodimers, which enhance the activation of M1-type microglia and inhibit M2-type microglia [37]. HMGB1 is an effective pro-inflammatory mediator to promote the polarization of M1-type microglia [38, 39] through TLR4 and TNF-α production [40]. Furthermore, activation of purinergic receptors, such as P2X7, triggers microglia proliferation and promotes the release of IL-1 and TNF-α [34].

### 3.1.1 M1/M2 phenotype in BBB damage and protection after AIS

M1-type microglia are implicated in cytotoxicity, superoxide generation, degranulation, and cytokine production [41]. For example, the pro-inflammatory cytokines interleukin-1β (IL-1β), interleukin-6 (IL-6), TNF-α, interferon (IFN), C-C chemokine ligand 2 (CCL2), and C-X-C motif chemokine ligand 10 (CXCL10), which are all produced by M1-type microglia, lead to oxidative stress, overproduction of nitric oxide (NO), and matrix metalloproteinase-9 (MMP-9) activation [42, 43]. These inflammatory cytokines are key mediators of BBB damage. When microglia release IL-1β and TNF-α, the BBB becomes more permeable and more likely to be damaged after AIS. For example, IL-1β enhances BBB permeability by downregulating ZO-1, occludin, and claudin-5 [44, 45], and TNF-α induces pericytes to release MMP-9, resulting in increased ECs permeability [46]. IFN-γ disrupts the integrity of the BBB by promoting cytoplasmic migration of ZO-1 and VE-cadherins from ECs [47]. In addition, chemokines CCL2 and CXCL10 secreted by M1-type microglia disrupt the BBB and increase the transport of immune cells to the brain parenchyma. The capacity of CCL2 in binding to the C-C chemokine receptor 2 receptor determines its effect on cerebral microvascular permeability [48]. Furthermore, elevated CCL2 release promotes monocytes and macrophages to migrate across the BBB, which exacerbates neurovascular unit injury and neuron loss [49]. In the permanent middle cerebral artery occlusion model, CXCL10 has been shown to increase monocyte and macrophage migration through the BBB [50].

The phagocytosis activity of microglia contributes to the removal of damaged tissues and tissue debris, thereby limiting the activation of danger-associated molecular pattern receptors and ultimately inhibiting the inflammatory response [51]. M2-type microglia secret a lot of anti-inflammatory cytokines, including IL-10, IL-4, IL-13, and transforming growth factor-β (TGF-β) [52]. These interleukins can directly promote the polarization of M2-type microglia cells. The transition from resting state to M2-anti-inflammatory type is mediated by interleukin-4 receptors (IL-4R), interleukin-10 receptors (IL-10R) [53], and vascular endothelial growth factor receptor 2 (VEGFR2) [54]. For example, in the ischemic penumbra of the MCAO model, neuron-derived IL-4 binds to IL-4R on the surface of neighboring microglia, causing these microglia to shift to the M2 phenotype and reducing ischemic brain injury [55]. Activation of IL-4R triggers phagocytosis mechanisms associated with tissue repair. Conversely, administration of SB431542, a TGF-β inhibitor, enhances microvascular permeability [56]. In addition, M2-type microglia produce growth and nutritional factors such as brain-derived neurotrophic factor (BDNF), vascular endothelial growth factor (VEGF), insulin-like growth factor 1 (IGF-1), and nerve growth factor [57].

**Figure 2.**
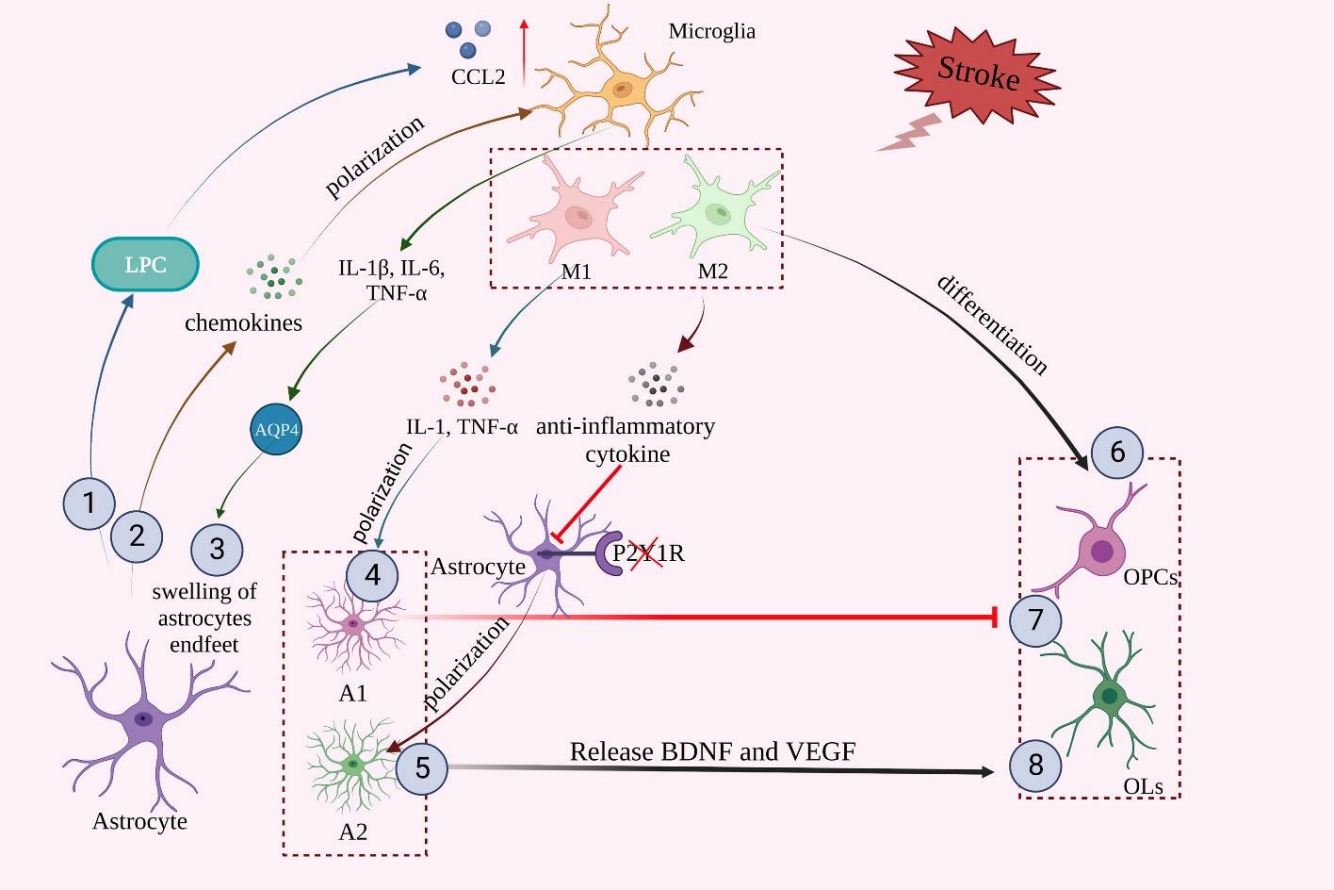
**Crosstalk of glial cells in the damage and protection of BBB after AIS**. ①. Lysophosphatidylcholine (LPC) released by astrocytes promotes microglia-induced C-C chemokine ligand 2 (CCL2) expression. ②. Chemokines released by astrocytes promote microglial polarization. ③. The inflammatory cytokines interleukin-1β (IL-1β), interleukin-6 (IL-6), and tumor necrosis factor-α (TNF-α) released by microglia induce upregulation of aquaporin 4 (AQP4), which leads to astrocyte endfeet swelling. ④,⑤. Pro-inflammatory cytokines released from M1 microglia and anti-inflammatory cytokines released from M2 microglia promote the polarization of astrocytes into A1/A2-type astrocytes. ⑥. M2 microglia promote the differentiation of oligodendrocyte progenitor cells (OPCs). ⑦. A1-type astrocytes can inhibit oligodendrogenesis and OPCs differentiation. ⑧. In contrast, A2-type astrocytes release anti-inflammatory cytokines and nutritional factors, such as brain-derived neurotrophic factor (BDNF) and vascular endothelial growth factor (VEGF), which promote oligodendrogenesis and OPCs differentiation.

### 3.1.2 Crosstalk between microglia and other glial cells (Fig. 2)

Microglia can regulate the immune process by inhibiting astrocytes, and they are essential for neuro-astrocyte crosstalk that occurs during the immune response after AIS [58]. Stroke-induced microglial activation can produce high levels of neurotoxic cytokines that in turn activate neighboring astrocytes. Current studies have shown that the inflammatory cytokines IL-1β, IL-6, and TNF-α released by microglia upregulate aquaporin 4 (AQP4), leading to the swelling of astrocytes endfeet [59]. Microglia can not only reduce OPCs number [60] but also promote OPCs differentiation [61]. The beneficial effect of microglia on OPCs is mainly due to the protective effect of M2 microglial polarization. Conditioned medium from LPS-induced M1-type microglia or untreated microglia increased OLs death 24 h after OGD treatment, and IL-4-conditioned M2-type microglia medium reduced OLs apoptosis [62]. M2-type microglia can drive OLs differentiation during remyelination after AIS [61].

### 3.2 Polarization of astrocytes and crosstalk between astrocytes and other glial cells

After AIS, astrocytes are activated and shift from resting to reactive state and reactive astrocytes upregulate many genes, increase the volume of the cytoskeleton, increase the expression and immunoreactivity of GFAP, and form glial scars [63]. Neuroinflammation and stroke produce two distinct types of reactive astrocytes, A1 pro-inflammatory and A2 anti-inflammatory [64]. A2-specific genes are dominant compared to A1-specific genes at 3 days after AIS [65] and phagocytic activity of astrocytes begins to increase on day 3 after AIS and continues for up to 14 days. Notably, reactive astrocytes engulf and degrade cell debris in the penumbra, inhibiting post-stroke inflammation by ATP-binding cassette transporter A1, and the absence of ATP-binding cassette transporter A1 significantly increases BBB permeability [66, 67].

### 3.2.1 A1/A2 phenotype in BBB damage and protection after AIS

Reactive astrocytes serve a dual role in maintaining BBB integrity after AIS. Resting A0-type astrocytes transformed into A1 phenotype by pro-inflammatory factors like IL-1α and TNF-α [64] and inhibition of A1-type astrocyte transformation by semaglutide could alleviate BBB destruction. By upregulating VEGF, IL-6, TNF-α, IL-1β and IL-15, as well as CCL2, CCL5, MMP, LCN-2 and ROS, astrocytes have a direct negative impact on the integrity of the BBB. For example, A1-type astrocyte-conditioned medium is known to reduce the expression of TJPs in ECs, suggesting that A1-type astrocytes could damage the integrity of BBB [36]. In addition, reduced TJPs expression in ECs caused by astrocyte-derived VEGF worsens BBB disruption, and neurological functional impairments [68]. Furthermore, polymerase δ-interacting protein 2 is also upregulated in poststroke astrocytes, leading to increased BBB leakage by inducing the expression of IL-6, CCL2, TNF-α, VEGF, and MMP [69]. More importantly, ROS can directly induce A1-type astrocytes, and microglia may aggravate the changes of A1-type astrocytes by releasing ROS [70]. A2-type astrocytes secrete IGF-1, IL-2, IL-10, and TGF-β to accelerate the anti-inflammation process. For example, IGF-1 produced by astrocytes maintains post-stroke integrity of BBB and neurological function after AIS by shifting immune cells toward an anti-inflammatory phenotype [71]. Notably, A2-type astrocytes can phagocytose other cells. In a rat model of photothrombotic stroke, increased expression of TGF-β and VEGF by A2-type astrocytes is accompanied by reduced BBB permeability, improved vascular damage, and increased angiogenesis [72]. AIS-induced A2-type astrocytes upregulate the expression of both neurotrophic and thrombin-sensitive proteins: the former promoting neuronal survival and growth while the latter promoting synaptic repair. A2-type astrocytes increase neurotrophins and cytokines, including cardiotrophin-like cytokine factor 1, LIF, IL-6, and IL-10, to support neuronal survival and repair [73]. For example, astrocyte-derived neurotrophins such as ciliary neurotrophic factor and glia-derived neurotrophic factor may help reduce acute inflammation and increase neuronal and OLs survival. Furthermore, ciliary neurotrophic factor can promote the activation of surrounding astrocytes in an autocrine/paracrine manner [74].

### 3.2.2 Crosstalk between astrocyte and other glial cells (Fig. 2)

AIS-induced astrocyte-derived BDNF not only promotes neuronal activity but also promotes OLs generation [75] and OPCs differentiation into mature OLs [76]. Conversely, reactive astrocytes, which may belong to the A1-type, block the proliferation and differentiation of OPCs [77]. Astrocytes release soluble substances that stimulate infiltration of peripheral immune cells, hence accelerating inflammation-induced BBB damage. By binding with chemokine receptors on microglia, astrocyte-derived chemokines have a paracrine effect on microglial polarization [68]. For example, astrocytes upregulate the expression of CX3CR1 and IL-4Rα on the surface of microglia through TGF-β, thereby inhibiting microglia activation [78]. In addition, lysophosphatidylcholine (LPC), a pro-inflammatory factor produced by neurons and astrocytes, stimulates microglia to upregulate the mRNA of inflammatory mediators CCL2 and CCR2 [49]. Furthermore, astrocytes-derived orosomucoid-2 can bind to C-C chemokine receptor type 5 (CCR5) of microglia to block C-X-C motif chemokine ligand 4 (CXCL4)-CCR5 interaction, which is the key of the anti-inflammatory effect of microglia [79]. After AIS, depletion of astrocyte-derived estrogen leads to reduced microglial activation and it can be reversed by exogenous 17β-estradiol [80].

### 3.3 Role of OPCs/oligodendrocytes in BBB damage and protection

### 3.3.1 Role of OPCs/oligodendrocytes in BBB damage

Previous studies have shown that OPCs can maintain the integrity of BBB and increase the expression of TJPs[81]. Thus, crosstalk between OPCs and ECs plays an important role in the formation and maintenance of the BBB. For example, Wnt/β-catenin pathway activated by OPCs plays important roles in the upregulation of TJPs after AIS. OPCs transplantation significantly reduces post-ischemic infarct volume and brain edema and maintains the integrity of the BBB [15]. OPCs may improve the recovery of neurological function by regulating angiogenesis and maintaining BBB integrity after AIS [15]. In addition, TGF-β1 from OPCs activates the MEK/ERK pathway in brain endothelial cells, thereby increasing TJPs expression and promoting BBB integrity [82]. Under physiological or pathological conditions, OLs release extracellular vesicles (EVs) which carry proteins such as myelin-associated proteins into neurons and EVs can improve the integrity of the BBB by regulating neurovascular coupling [83].

### 3.3.2 Role of inflammatory response in OPCs/oligodendrocytes-induced BBB damage after AIS

After AIS, OLs impairment produces large amounts of ROS [28], resulting in demyelination and brain injury [84]. OPCs-conditioned medium can induce TJPs expression and reduce BBB permeability by activating TGF-β-receptor-MEK/ERK signaling pathway[85]. When WM is damaged, inflammation and oxidative stress stimulate a significant increase in MMP-9 release from OLs and OPCs and infiltration of neutrophils into the mice brain, leading to the destruction of vascular structure and inhibiting the repair of the BBB [85].

Microglia release IL-1β and IFN-γ, and IFN-γ promotes OLs while inhibiting OPC proliferation [86]. More importantly, IL-4 stimulates OLs regeneration and remyelination through the IL-4/PPARγ signaling axis [87]. After stroke-induced white matter (WM) injury, regulatory T cells (Tregs) secrete IL-6 and FGF-2 and promote OPCs differentiation [88]. More importantly, peripheral immune cells are also involved in post-stroke inflammation and interact with OPCs and OLs. After AIS, BBB breakdown causes OLs antigens such as myelin oligodendrocyte glycoprotein (MOG) and myelin basic protein to leak into the peri-infarction area [89]. MOG increases infarct volume by promoting the infiltration of MOG-responsive spleen cells into the lesion area [89]. In addition, VEGF-A produced by activated T cells has been shown to increase OPCs proliferation by activating VEGFR2 [90].

### 3.4 Role of pericytes in BBB damage and protection

Pericytes maintain the integrity of the BBB by regulating TJPs formation and transport across endothelial vesicles. More importantly, pericytes inhibit the expression of molecules that increase vascular permeability during CNS immune cell infiltration and BBB development [91]. Under hypoxic conditions, pericytes enhance a tighter barrier function and protecte the ECs from hypoxic damage [92]. On the other hand, early detachment of pericytes from the cerebral microvasculature also leads to loss of TJPs and BBB leakage [93]. More importantly, pro-inflammatory cytokine IL-6 in pericytes is known to be produced soon after the onset of AIS and the expression of microglial markers to obtain a microglial phenotype, thus participating in the inflammatory response to AIS [94].

### 3.5 Role of glial cells and endothelial cells crosstalk in the injury and protection of BBB after AIS.

Angiogenesis can restore blood flow and oxygen supply in ischemic tissue and is the basis of ischemic brain repair[95]. Angiogenesis is closely associated with prolonged survival and BBB integrity of stroke patients.

Shortly after AIS, microglia activated by neuronal death release ROS, MMPs, and chemokines (TNFα, IL-1α, IL-1β, CCL2, and CXCL10), inducing ECs activation and BBB disruption [31]. For example, TNF-α decreases the expression of claudin-5 and ZO-1 on ECs and induces EC necroptosis [11, 96]. TNF-α activates ECs to express pro-inflammatory cytokines and releases MMP-9, TJPs. As a result, ECM proteins are degraded, and BBB is damaged [97]. CCL2 and CXCL10 can exacerbate BBB disruption by promoting the entry of peripheral immune cells into the CNS [98]. Of note, ECs are also able to suppress the pro-inflammatory phenotype of microglia in the chronic phase of AIS. For example, VEGF secretion by ECs significantly reduces the expression of pro-inflammatory cytokines and iNOS in microglia [99]. ECs-derived NO and the immunosuppressive molecule CD200 on the surface of brain ECs inhibit microglia activation and reduce inflammation [100]. In addition, MMPs and VEGF secreted by astrocytes also increase vascular permeability and vasogenic edema after AIS[101], thereby aggravating BBB damage, infarct progression, and neurological dysfunction [68]. Increased VEGF-A expression in reactive astrocytes leads to BBB breakdown via downregulating claudin-5 and occludin in ECs [102]. Astrocytes also release soluble substances that activate microglia and recruit peripheral immune cells, which indirectly increases the inflammation-induced BBB breakdown [103].

BMECs-derived platelet-derived growth factor-BB triggers the secretion of TGF-β from OPCs through the activation of PDGFRα/PI3K/Akt signaling pathways [22]. Thus, crosstalk between OPCs and BMECs plays an important role in the formation and integrity of the BBB through their secretion factors. In addition to OPCs, the presence of OLs has been reported to reduce the permeability of BMECs through unknown soluble factors [104].

**Figure 3.**
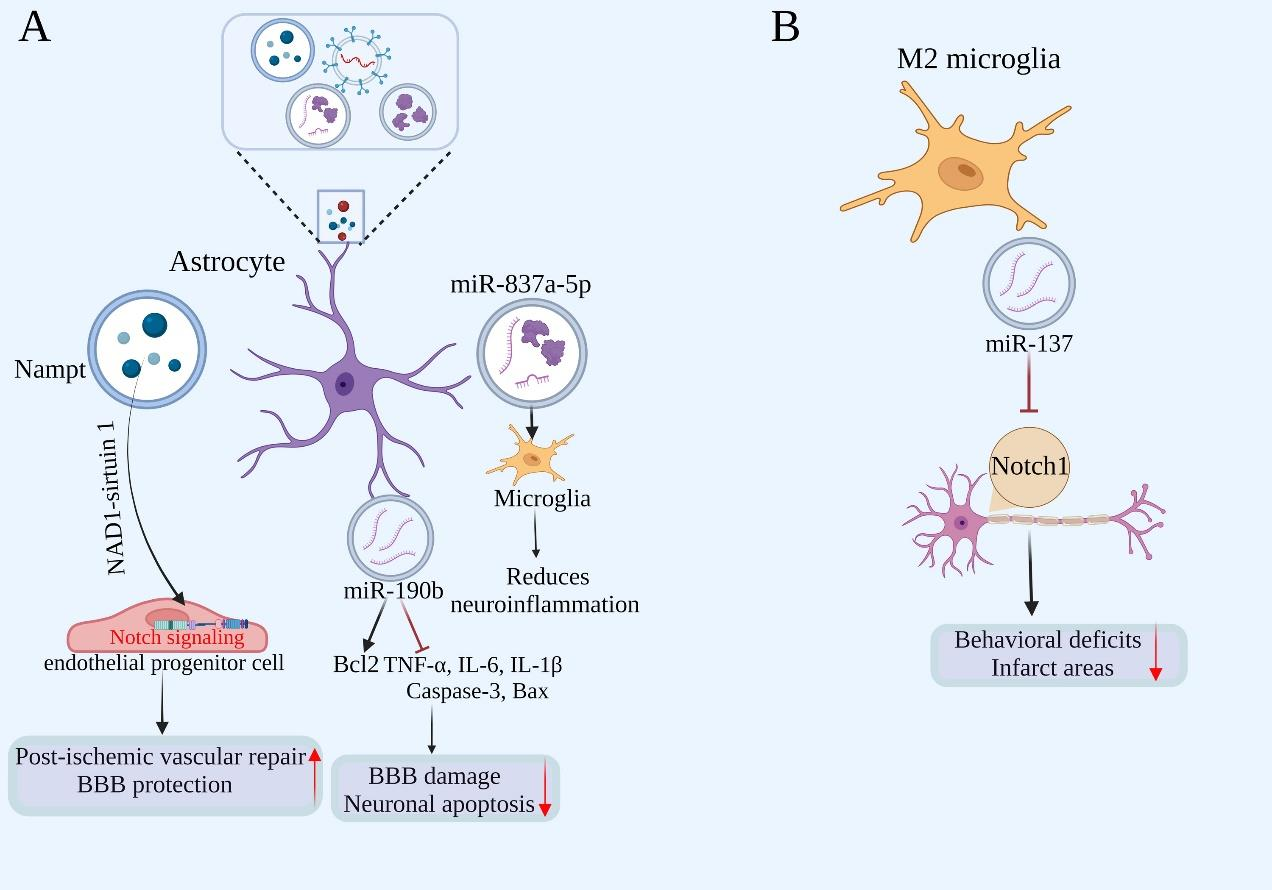
**Glial cell-derived exosomes and the BBB after AIS**. A) The Nampt-NAD1-sirtuin 1 cascade improves post-ischemic vascular repair by modulating Notch signaling in endothelial progenitor cells. MiR-190b alleviates neuronal apoptosis and BBB damage by up-regulating the expression of B-cell lymphoma-2 (Bcl2) and down-regulating the expression of pro-inflammatory factors. MiR-837a-5p regulates microglial polarization in damaged neurons to reduce neuroinflammation. B) M2 microglia-derived miR-137 reduces behavioral deficits and infarct areas by inhibiting Notch1 signaling in neurons.

## 4. Role of glial cell-derived exosomes in BBB damage and protection after AIS (Fig. 3)

### 4.1 Role of astrocyte-derived exosomes in BBB damage and protection after AIS

Exosomes are EVs formed by plasma membrane fusion [105], which are important intercellular communication mediators released by CNS cells and can be a promising strategy to treat AIS[106]. Previous studies have shown that exosomes can transport a variety of microRNAs (miRNAs) and proteins, which can promote angiogenesis, anti-inflammatory and anti-apoptosis after AIS [107, 108].

Under pathological and aging conditions, activated astrocytes and microglia influence the function and phenotype of other cell types through secretion of EVs [109]. Astrocyte-derived extracellular vesicles (ADEVs) not only regulate neighboring CNS cells but also participate in communication between the CNS and peripheral immune cells. IL-1β or TNF-α can promote proteins and miRNAs in ADEVs to cross BBB to promote peripheral acute cytokine response [83, 110]. For example, ADEVs enriched in miR-873a-5p can alleviate neuroinflammation through microglia phenotypic regulation after brain injury [111]. In addition, ADEVs appear to significantly inhibit OGD-HT-22 cell apoptosis and reduce TNF-α, IL-6, and IL-1β levels, indicating a beneficial effect of ADEVs on BBB damage-associated brain edema and hemorrhage after AIS. Moreover, nicotinamide phosphoribosyltransferase (Nampt), the rate-limiting enzyme for nicotinamide adenine dinucleotide synthesis in mammals, can also be secreted by astrocytes and found in ADEVs [112]. Clinical studies have confirmed that the mean plasma concentration of Nampt in ischemic stroke patients is 2-8 times higher than the Ctrl [113]. The Nampt-NAD1-sirtuin 1 cascade improves post-ischemic vascular repair by modulating Notch signaling in endothelial progenitor cells, which may play a role in BBB protection after AIS [114].

### 4.2 Role of microglial cell-derived exosomes in BBB damage and protection after AIS

Exosomes generated from M2-type microglia (BV2-Exo) play a crucial role in mediating the neuroprotective effects of BV2 cell conditioned medium. Exosomes released by microglia BV2 cells in the M2 phenotype are absorbed by ischemia-injured neurons, boosting ischemic neuron survival via the Notch1 pathway. Furthermore, knockdown of miRNA-137 partially reverses the neuroprotective effects of BV2-Exo, suggesting that miRNA-137 is involved in the beneficial role of microglia exosomes [115].

## 5. Crosstalk between peripheral immune cells and glial cells after AIS (Fig. 4)

The inflammatory response after AIS is not only limited to resident immune cells but also involves the infiltration of peripheral immune cells into the brain parenchyma. Peripheral immune cells are a potential target for AIS treatment because they respond earlier to ischemic damage and are more accessible than cells from the CNS. Removal of microglia has been reported to increase the infiltration of neutrophils, macrophages, T cells, and NK cells in the brain [116]. Cerebral immune cells regulate the recruitment, extravasation, and function of peripheral immune cells. After ischemic injury, the number of microglia, astrocytes, and pericytes upregulate and release different chemokines to stimulate neutrophils, monocytes, and lymphocytes to infiltrate the ischemic hemisphere [103].

### 5.1 Crosstalk between T cells and glial cells after AIS

After AIS, CD8^+^ cytotoxic T cells are the first immune cell type to invade the brain, resulting in neuron death and aggravated brain injury by releasing perforin/granzyme [117]. Microglia and astrocyte-derived IL-15 can increase the number and activity of CD8^+^ T cells and NK cells, thus exacerbating the destruction of BBB [118]. Activated microglia can stimulate CD4^+^ T cells to differentiate into Th1 or Th2 cells, which in turn produce pro-inflammatory or anti-inflammatory cytokines that damage or protect the brain [119]. Th1 subpopulations promote M1 polarization of microglia by secreting pro-inflammatory factors such as IFN-γ, which accelerates the amplification of inflammation. M1-type microglia promote Th1 cell polarization by secreting IL-12 and TNF-α in the early stage after AIS. Th2-derived IL-4 inhibits NF-κB expression and NLRP3 formation in HMGB-1-induced astrocytes activation via STAT6/PPARγ and enhances M2 polarization of microglia and phagocytosis in microglia through IL-4 secretion after AIS [120].

During the chronic phase after AIS, the number of Treg in the brain increase significantly, and they contributed to the neurological functional recovery [121]. In addition, microglia inhibit the anti-inflammatory effect of Tregs by inducing HIF-1α/Sirtuin2 overexpression in Tregs via intercellular contact [122] and HIF-1α has been shown to contribute critically to BBB damage after AIS [123, 124]. Tregs reduce microglia activation by releasing IL-4, IL-10, and TGF-β [125]. When Tregs are absent, microglia convert to the M1 phenotype, and if Tregs are increased, microglia convert to the M2 phenotype. Tregs play an important role in promoting the development of M2-type microglia by up-regulating the expression of IL-10-mediated glycogen synthetase kinase 3β [126]. Activation of peripheral Tregs is thought to represent an endogenous mechanism for reducing brain inflammation after AIS [127]. Twelve weeks after AIS, transmigrated Tregs ameliorate BBB disruption through regulating MMP-9, and the specific mechanism of action may involve the programmed death-1 ligand 1 (PD-L1). PD-L1 has been shown to bind to programmed death-1 (PD-1), delivering inhibitory signals that lead to suppression of the immune response [128]. Twenty-four hours after MCAO, PD-L1 deficiency eliminates the protection effect of Tregs on BBB integrity [129]. In addition, Tregs inhibit CCL2 production in ECs following ischemia and tPA treatment. Notably, Treg therapy appears to be able to exert an additional protective effect in CCL2 knockout mice, maintaining BBB integrity and reducing the risk of tPA-induced HT after AIS through Tregs-dependent dual inhibition of MMP-9 and CCL2 [130]. CCR5^-/-^ Treg expression deficiency significantly increases BBB leakage, and knockdown of CCR5 in Tregs significantly eliminates their protective effect on BBB. In addition, CCR5 signaling promotes Tregs accumulation and enhances the inhibitory effect of transferred Tregs on neutrophil-derived MMP-9. Furthermore, pretreatment with the CCR5 inhibitor D-ala-peptide T-amide reduces Treg-mediated protection against BBB leakage [131]. RNA-seq analysis of microglial co-cultures with activated Tregs has shown significant upregulation of genes associated with the anti-inflammatory phenotype and genes encoding proteins related to brain injury or repair. In addition, cytokines such as CXCL2 and FGF1 were upregulated in Tregs-stimulated microglial. Treg-microglial interactions generate an osteopontin-rich microenvironment to optimize microglia responses, promote OLs regeneration, and facilitate WM repair [121].

**Figure 4.**
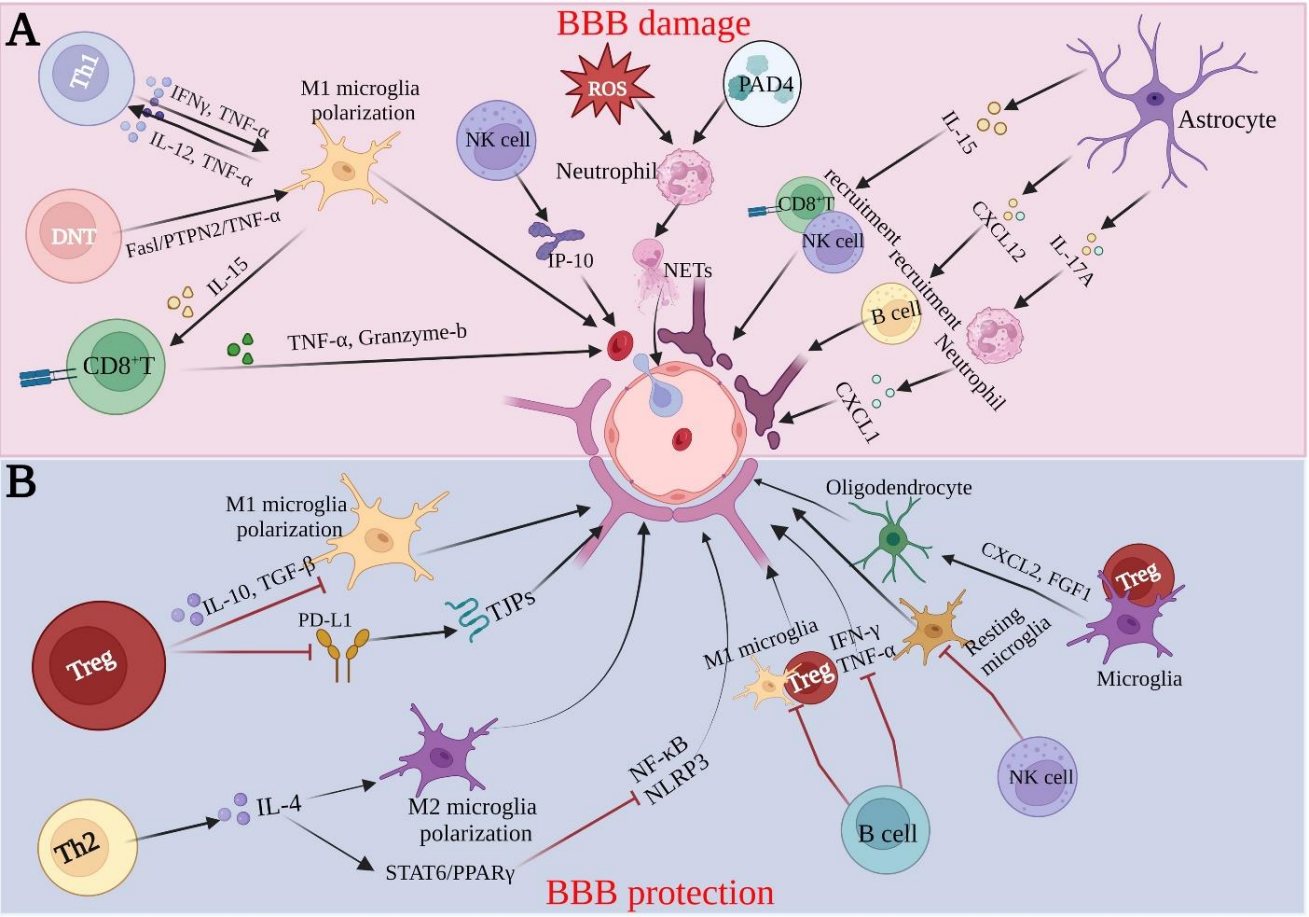
**Crosstalk between glial cells and peripheral immune cells regulating BBB integrity after AIS. (A)** Pro-inflammatory cytokines promote interaction and polarization between Th1 and M1 microglia, causing BBB injury. Double-negative T cells (DNT) increase the number of M1 microglia via the Fas ligand/protein tyrosine phosphatase non-receptor type 2/tumor necrosis factor-α pathway. M1 microglia enhance CD8^+^T activity by secreting interleukin-15 (IL-15), and conversely, CD8^+^T cell induces cytotoxicity via granzyme-b and tumor necrosis factor-α (TNF-α), enhancing stroke progression. NK cells can damage BBB via interferon-inducible protein-10 (IP-10). Reactive oxygen species (ROS) and peptidylarginine deiminase 4 (PAD4) stimulate neutrophils to produce neutrophil extracellular traps (NETs) and astrocytes to secrete pro-inflammatory cytokines, such as IL-15 and interleukin-17A (IL-17A) and secrete chemokines to promote the recruitment of CD8^+^T cells, NK cells and B cells, resulting in BBB damage. **(B)** Treg enhances tight junctions (TJPs) of BBB and protection by both inhibiting M1 microglia polarization and enhancing TJP expression. Th2 protects the BBB by promoting M2 microglia polarization with inhibition of NF-κB and NLRP3-mediated inflammatory responses. B cells inhibit the post-ischemic response of M1 microglia with Treg. Cytokines C-X-C motif chemokine ligand 2 (CXCL2) and fibroblast growth factors 1 (FGF1) are upregulated in Treg-stimulated microglia, promote Treg-microglia interaction and oligodendrogenesis, and promote white matter repair, thereby protecting the BBB.

### 5.2 Crosstalk between B cells and glial cells after AIS

Astrocytes can produce TNF family proteins, activators of the B cell that are involved in B cell development, survival, and function [132]. B cells reduce the volume of infarcts and neurological deficits by reducing the post-ischemic response of other immune cells, such as T cells, macrophages, and microglia [133]. B cells can also suppress the production of cytokines like IFN-γ and TNF-α, as well as the infiltration of inflammatory T cells [134]. Cerebral ischemia-induced spleen atrophy is associated with a decrease in the numbers of inflammatory cytokines, T cells, and B cells [135]. On the other hand, B cells may cause delayed cognitive impairment after AIS, hindering long-term recovery[136]. LPS preconditioning increases the amount of regulatory B cells (Bregs) in the spleen, initiates the anti-inflammatory protective mechanism of the spleen, and decreases the damage to the BBB after AIS [137].

### 5.3 Crosstalk between neutrophil and glial cells after AIS

Fourteen days after AIS, neutrophil depletion reduces the damage of the BBB [138]. Neutrophils produce intravascular and intraparenchymal neutrophil extracellular traps (NETs) after AIS [139]. Peptidylarginine deiminase 4 (PAD4), one factor affecting NETs formation, is significantly upregulated in the ischemic hemisphere of tPA-treated mice, and BBB permeability is significantly increased with PAD4 overexpression. Moreover, DNase I inhibition or PAD4 knockdown significantly inhibits NETs production, thereby enhancing the expression of TJPs and adhesion junction protein VE-cadherin in brain microvessels, demonstrating that neutrophil NETs play a critical role in post-stroke vascular remodeling and BBB maintenance [138]. tPA treatment increases NETs formation in mice with thrombotic stroke. In addition, NETs appear to be associated with increased BBB damage, and increased cerebral hemorrhage in tPA-treated mice, which can be mitigated by the use of DNase I to clear NETs. Activation of cGAS-STING pathway and the production of IFN-β seem to participate in the NETs-mediated tPA’s side effect. Thus, NETs are a key target for promoting stroke-mediated angiogenesis and the resulting functional recovery [140]. ROS can be released by neutrophils that exert deleterious effects on ZO-1 [141] and BMs to destroy the structure of the BBB [142]. Neutrophils can also produce neutrophil-derived cytokines such as IL-1β, IL-6, TNF-α, and CCL2, which disrupt the integrity of the BBB [143].

Recent studies have shown that neutrophils may exhibit anti-inflammatory phenotype by expressing Ym1 and CD206. While being hindered by TLR activation, PPARγ agonists accelerate N2 polarization of neutrophils and promote phagocytosis of neutrophils by microglia, thereby reducing brain edema and infarct volume [144]. After AIS, astrocytes attract neutrophils by increasing C-X-C motif chemokine ligand 1 secretion in response to the synergistic action of TNF-α and IL-17A [145].

### 5.4 Crosstalk between NK cells and glial cells after AIS

NK cells infiltrate the peri-infarction area of the ischemic hemisphere after AIS in humans. They catalyze neuronal death through perforin/granase apoptotic pathway and accelerate cerebral infarction [146]. In addition, NK cells can also damage BBB via interferon-inducible protein-10 (IP-10), and neutralization of IP-10 can prevent the release of IFN-γ and other cytotoxic cytokines, and reduce NK-cell-mediated damage after AIS [50]. NK cells kill resting microglia with the natural killer subgroup cells expressing NKG2D receptor and natural cytotoxicity receptor NKP46. Under inflammatory conditions, astrocytes produce different soluble mediators that stimulate or inhibit NK cell activity, depending on their properties and location. For example, during ischemia, astrocytes are a major source of IL-15, which activates NK cells [147].

## 6. Glial cells could be a therapeutic target for alleviating BBB damage after AIS (Table 1)

### 6.1 Inhibition of TLR and NF-κB expression

TLR2 and TLR4 mediate the microglia activation-produced pro-inflammatory cytokines and brain injury after AIS [34, 148], and TLR2 or TLR4 deficiency reduces the production of TNF-α, iNOS, and COX2, and decrease infarct volume [149]. In addition, when TLR is activated, microglia upregulate the IL-17-specific receptor IL-17RA and uniquely produce more IL-17, leading to neuronal damage [150]. Therefore, the TLR2/TLR4/IL-17 pathway may be a potential target to inhibit inflammatory response and improve neurological prognosis after AIS. Of note, inhibition of M1-type microglia activation and enhancement of M2-type microglia activation through the TLR4/NF-κB pathway is essential for the recovery of AIS patients [36].

TLR/NF-κB signaling is the first step in astrocyte activation. The expression of TLR and NF-κB can be inhibited by cottonseed oil [151], Z-guggulsterone [152], IL-32a [153], microRNA-1906 [154], ginkgoaceae [155] or ligustilide [156]. In addition, natural (poly) phenols can not only promote M2-type polarization of microglia, but also reduce neuroinflammation and the number of reactive astrocytes and the levels of inflammatory cytokines, resulting in a significant reduction of BBB disruption, neurological dysfunction, and brain edema [36].

### 6.2. Inhibition of NLRP3

NLRP3 expression in microglia is known to reach a peak 24 h after reperfusion, and NLRP3 is highly expressed and induces the production of pro-inflammatory mediators, which also activated microglia-mediated neurotoxicity, leading to neuronal death and BBB damage. In addition, NLRP3 also helps to up-regulate MMP-2/9, reduce TJPs, and increase the permeability of ECs. Moreover, the protective effect of NLRP3 inhibition appears to be eliminated in mice lacking microglia or Gr-1+ myeloid cells [157]. MCC950, an inhibitor of NLRP3, can block NLRP3 activation, improve the BBB after intracerebral hemorrhage and reduce brain injury [17]. Minocycline, a member of the tetracycline family of antibiotics, inhibits the activation of microglia and promotes M2-type microglia polarization by inhibiting NLRP3 [158]. Minocycline treatment can reduce cerebral infarct volume, tissue loss, and neurological dysfunction, and significantly alleviate BBB destruction and cerebral hemorrhage in mice after AIS [159]. Many NLRP3 inhibitors, such as adiponectin [160], telmisartan [161], MCC950 [162], and sinomenine (SINO) alleviate A1-type astrocyte-induced BBB disruption and neurological dysfunction by inhibiting NLRP3. For example, Telmisartan treatment dose-dependently improves AIS-induced NLRP3 inflammasome activation in hypertensive rats, significantly reduces amyloid-β deposition in neurons, and may have a preventative effect on the development of Alzheimer's disease pathology in the brain by alleviating the NLRP3 inflammasome after AIS [161, 163]. In addition, SINO treatment significantly reduces cerebral infarction, cerebral edema, neuronal apoptosis, and neurological dysfunction. SINO also inhibits inflammation by inhibiting NLRP3 activation through an AMPK-dependent pathway [164].

**Table1:** Targeting glial cells for treatment of BBB damage after AIS.

Molecular targets	Drug/RNA	Cell	Outcomes	Reference
**TLR2/TLR4/NF-κB**	Cottonseed oil	Microglial/Astrocytes	Reduced BBB damage, infarct volume and bleeding	[148]
	Z-Guggulsterone		Reduced infarct volume and neurological deficit	[149]
	IL-32α			[150]
	MicroRNA-1906			[151]
	Ginkgoaceae		Reduced BBB leakage, infarct volume and neurological prognosis	[152]
	Ligustilide		Reduced cerebral infarct volume and loss of nerve function	[153]
**NLRP3**	MCC950	A1-type astrocytes	Improved the integrity of BBB and reduce brain injury	[17, 159]
	Minocycline	M2-type microglial	Reduced BBB damage, infarct volume and bleeding	[155]
	Adiponectin	A1-type astrocytes	Alleviated the brain edema, neuronal apoptosis, and neurological deficiency	[157]
	Telmisartan	A1-type astrocytes	Reduced inflammation and Protected NVU	[158]
	Sinomenine	A1-type astrocytes	Alleviated the brain edema, neuronal apoptosis, and neurological deficiency	[161]
**RNA**	miR-29b	Astrocytes	Reduced edema, infarct volume and BBB destruction	[166]
	miR146a	Astrocytes	Reduced the release of pro-inflammatory cytokines	[167]
	LncRNA H19	Microglial/Astrocytes	Reduced BBB damage	[169, 170]

### 6.3 ANXA2

Annexin has been shown to play a protective role after AIS [165]. For example, annexin A2 (ANXA2) gene knockout results in decreased expression of the BBB TJPs and increased BBB permeability [165]. ANXA2's protective effects on microglia and the BBB are mediated by formyl peptide receptors and the Robo4-paxillin-ADP ribosylation factor pathway. ANXA2 also regulates pro-inflammatory response signaling and ROS production in microglia by regulating IL-17 [166].

### 6.4 RNAs

Changes in miRNA expression profiles of the major cell types of the CNS suggest that miRNAs may be involved in ischemic injury [167]. During hypoxic injury, miR-210 is significantly upregulated in astrocytes and activated by HIF-1α [168]. In addition, miR-29b in the brain and blood are both downregulated in ischemic mice, and dual-luciferase reporter system has shown that AQP4 is the direct target of miR-29b. Overexpression of miR-29b can decrease AQP4 expression, infarct volume, and BBB damage [169]. MIR-146a is rich in astrocytes and can effectively regulate inflammatory response through interaction with TLR signals [170], and can inhibit IRAK-1, TRAF-6, and pro-inflammatory cytokines release [171]. LncRNA H19, one of the most widely studied lncRNAs [172], is positively correlated with plasma claudin-5 and MMP-9 levels in patients with AIS and correlated with the extent of BBB destruction. In addition, lncRNA H19 levels have been found to induce phenotypic polarization of M1-type microglia to M2-type microglia by inhibiting the phosphorylation of microglia HDAC1 protein and regulating neurogenesis through Notch/P53 pathway. Moreover, lncRNA H19 is transported from neurons to astrocytes, thereby inhibiting the expression of miR-18a in astrocytes and inducing an increase in the production and secretion of VEGF, and TJPs reduction in endothelial cells [173].

### 6.5 Other targets and strategies

AS605240, a PI3Kγ inhibitor, significantly reduces astrocyte activation and pro-inflammatory cytokine expression, thereby improving stroke prognosis [174]. Oleoylethanolamide, a bioactive lipid mediator, promotes the expression and nuclear transport of PPARα in astrocytes to inhibit activation and neural loss of astrocytes in the ischemic hemisphere [175]. Memantine, an N-methyl-d-aspartate receptor antagonist, reduces BBB leakage and infarct extent by reducing astrocyte-induced MMP2/9 expression [176]. Melatonin is a hormone secreted by the pineal gland, and has antioxidant effects, alleviating the ischemia reperfusion-induced BBB breakdown by regulating α7nACh receptors and clearing ROS produced by glial cells after AIS [39, 177].

## 7. Conclusion

Crosstalk between glial cells plays a critical role in the damage and protection of BBB after AIS. In addition, polarization, inflammatory response of glia cells and glial cell-derived exosomes are key contributory factors to the damage and protection of BBB after AIS. Finally, crosstalk between glial cells and immune cells is implicated in the damage and protection of BBB after AIS, therefore regulating the crosstalk between glial cells and immune cells would hold the promise to alleviate AIS-induced BBB damage.
